# High parasitism by *Primasubulura jacchi* (Ascaridida: Subuluridae) and infestation of *Rhipicephalus sanguineus* sensu lato (Acari: Ixodidae) in *Callithrix jacchus* (Primates: Callitrichidae) in Northeastern Brazil

**DOI:** 10.1007/s11259-026-11316-y

**Published:** 2026-06-11

**Authors:** Eduardo Henrique Amorim Silva, Eduardo Fernandes da Silva, Ananda Maria Freitas Freire Leão, Anna Cecília Oliveira Santos, Tatiene Rossana Móta Silva, Márcia Bersane Araújo de Medeiros Torres, Ruben Horn Vasconcellos, Lucia Oliveira Macedo, Rafael Antonio Nascimento Ramos, Gílcia Aparecida de Carvalho

**Affiliations:** 1 Laboratory of Parasitology, Federal University of the Agreste of Pernambuco, Garanhuns, Pernambuco Brazil; 2 Laboratory of Animal Anatomy and Pathology, Federal University of the Agreste of Pernambuco, Garanhuns, Pernambuco Brazil; 3 Graduate Program in Animal Bioscience, Federal Rural University of Pernambuco, Recife, Pernambuco Brazil; 4 Department of Veterinary Pathobiology, College of Veterinary Medicine & Biomedical Sciences, Texas A&M University, College Station, TX USA; 5 Department of Biology, Federal Rural University of Pernambuco, Recife, Pernambuco Brazil

**Keywords:** common marmoset, tick, helminth, anthropization, parasitic fauna

## Abstract

The common marmoset (*Callithrix jacchus*) is a neotropical primate widely distributed in Brazil and frequently associated with urban and peri-urban environments, a condition that favors exposure to different parasites. The present study aimed to report the occurrence of *Primasubulura jacchi* and to document the first record of *Rhipicephalus sanguineus* sensu lato parasitizing *C. jacchus* in Northeastern Brazil. A debilitated female *C. jacchus* was rescued in a peri-urban area of the municipality of Garanhuns, Pernambuco, and referred to the Veterinary Hospital of the Federal University of the Agreste of Pernambuco for clinical evaluation. Due to the severe clinical condition, the animal was euthanized. During necropsy, a fecal sample was collected directly from the rectum and analysed through egg flotation technique. Main macroscopic findings included extensive cutaneous lesions on the left thoracic and pelvic limbs, intense tick infestation and intestinal parasitism by nematodes. Ticks were morphologically identified as *Rh. sanguineus* s.l., whereas adult nematodes and eggs were identified as *P. jacchi*. This study expands the knowledge of the parasitic fauna associated with *C. jacchus* in Northeastern Brazil and reinforces the importance of health monitoring of primates in anthropized environments.

**Supplementary Information:**

The online version contains supplementary material available at https://doi.org/10.1007/s11259-026-11316-y.

## Introduction

Neotropical primates play a critical role in biodiversity and conservation through the maintainance of the ecological balance of forest ecosystems, especially in remnant forest areas such as the Atlantic Forest fragments in Brazil (Silva et al. 2018). These animals act primarily as important seed dispersers and regulators of invertebrate populations, contributing to vegetation dynamics and regeneration (Rylands et al. 2009; Bufalo et al. 2016; Silva et al. 2018). Among these primates, marmosets of the genus *Callithrix* are distributed across the Atlantic Forest and Cerrado biomes, encompassing the Northeast, Southeast, and Central-West regions of Brazil, with six recognized species (*Callithrix jacchus*, *Callithrix aurita*, *Callithrix penicillata*, *Callithrix flaviceps*, *Callithrix geoffroyi*, and *Callithrix kuhlii*), as well as hybrid populations reported in these areas (Rylands et al. 2009).

Despite their ecological relevance, primates have been directly impacted by intense habitat fragmentation associated with increasing urban expansion, which are factors that increase their exposure to infectious and parasitic agents and alter host-parasite dynamics (Thatcher et al. 2021). Certain marmoset species exhibit high ecological plasticity, enabling them to occupy and adapt to anthropized environments. *Callithrix jacchus* and *C. penicillata* stand out due to their growing occurrence under synanthropic conditions, including fully urbanized areas, where contact with humans and domestic animals is more frequent (Pinheiro and Pontes 2015; Andrade 2022; Dias et al. 2022).

As a consequence of this urbanization process and ecological adaptation, *C. jacchus* and *C. penicillata* rank among the main marmoset species in urbanized environments presenting a diverse parasite fauna (Silva et al. 2018; Dias et al. 2022, 2024; Santos et al. 2025). These records, mostly originating from Southeastern Brazil, include protozoa such as *Cryptosporidium* spp., *Giardia* spp. and *Leishmania* spp. (Paiz et al. 2019; Lima et al. 2021), helminths represented by genera such as *Primasubulura*, *Subulura*, *Ancylostoma*, and *Strongyloides* (Corrêa et al. 2016; Lima et al. 2021; Dias et al. 2024), and arthropods including ticks of the genera *Amblyomma* and *Haemaphysalis* (Martins et al. 2021). Nematodes of the family Subuluridae, with emphasis on species of the genera *Primasubulura* and *Subulura*, are among the most frequently described parasites in *C. jacchus* and its hybrids (Dias et al. 2024).

Although the genus *Callithrix* has a wide geographic distribution in Brazil, studies focused on characterizing the parasitic fauna of these marmosets, especially ectoparasites, remain scarce in the Northeastern region of the country (Rylands et al. 2009; Dias et al. 2024). The knowledge on the occurrence of helminths and ectoparasites in *C. jacchus* in this region is essential to expand understanding of the parasitic diversity associated with the species and to document new records of helminths and ectoparasites in urban and peri-urban areas.

Furthermore, the occurrence of ectoparasites, such as *Rhipicephalus sanguineus* s.l., parasitizing hybrids of *Callithrix* spp., has previously been reported in Brazil, particularly in the Southeast region (Martins et al. 2021). This tick species is commonly associated with urban environments and domestic dogs, and its parasitism in primates highlights the relevance of these findings for understanding interactions between wildlife and anthropized environments (Dantas-Torres 2010). In this study, we report the occurrence of *P. jacchi* and the first record of *Rh. sanguineus* s.l. in *C. jacchus* in Northeastern Brazil.

## Case description

### Clinical presentation and procedures

A female *C. jacchus* popularly known as the common marmoset, was rescued by the *Agência Estadual de Meio Ambiente* (*CPRH*) in a peri-urban area of the municipality of Garanhuns (8°52’28.41"S, 36°28’06.22"W), state of Pernambuco, Brazil, and referred to the Veterinary Hospital of the Federal University of the Agreste of Pernambuco (UFAPE) for clinical evaluation.

During physical examination, the animal presented marked debility, low body condition score, dehydration, pale mucous membranes, extensive lesions with necrotic areas on the left thoracic and pelvic limbs, and intense tick infestation. Due to the severe clinical condition and the infeasibility of rehabilitation and reintroduction into the wild environment, euthanasia was performed in accordance with current ethical and legal protocols (CFMV 2012). The animal subsequently underwent necropsy, and macroscopic findings were properly documented through photographic records.

During necropsy, ticks were manually removed using anatomical forceps and stored in vials containing 70% ethanol. Morphological identification was performed under a stereomicroscope using anatomical characters and specific dichotomous and pictorial keys for the family Ixodidae (Walker et al. 2000; Dantas-Torres et al. 2013). Additionally, helminths were observed in the small intestine of the specimen, which were collected and subjected to morphological identification using taxonomic keys for nematodes (Vicente et al. 1997; Anderson et al. 2009).

Fecal samples obtained directly from the large intestine content were processed using the centrifugal flotation technique in zinc sulfate solution (Zajac et al. 2002). Slides were stained with Lugol’s iodine and examined under a binocular light microscope (Foreyt 2013). Parasitic structures were photographed using a digital camera attached to the microscope, and the images were morphometrically analyzed using T Capture software.

### Parasitological findings

**Fig. 1:**
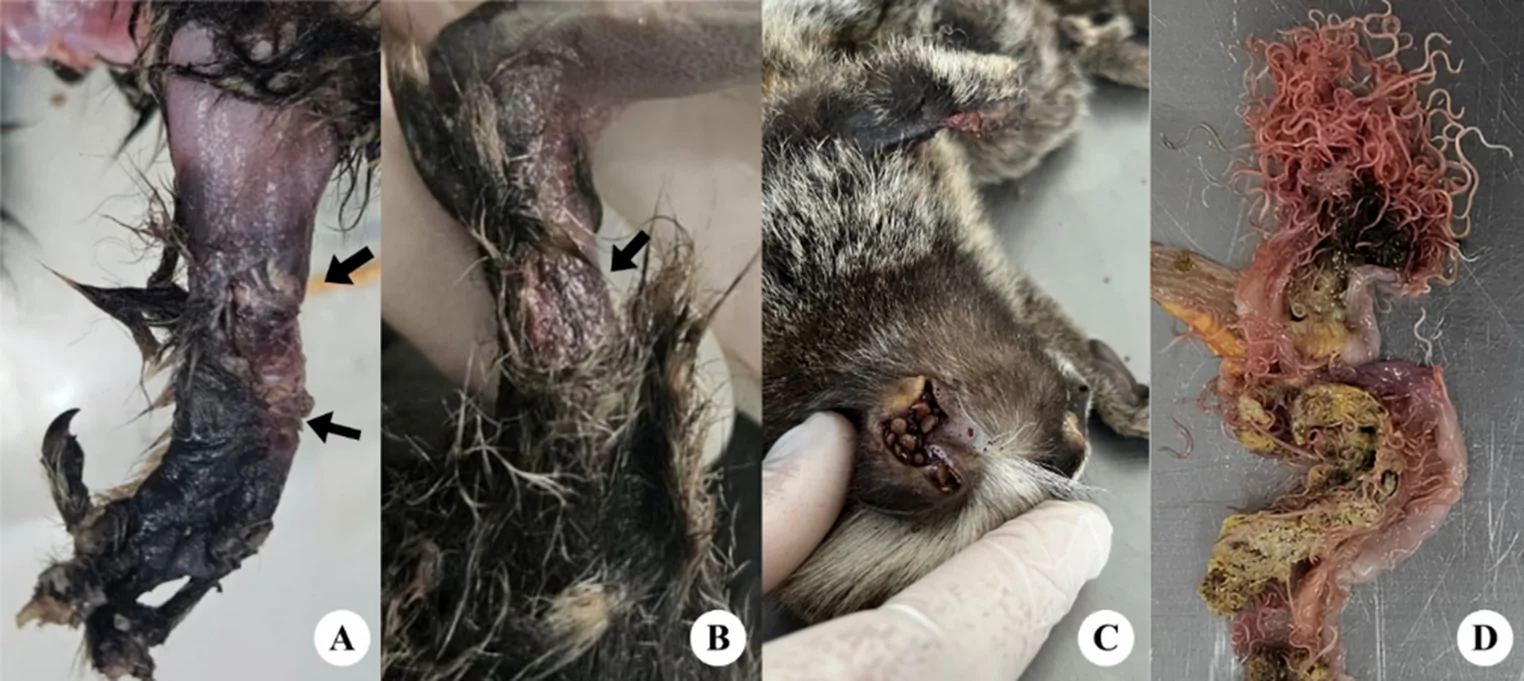
Macroscopic findings observed in *Callithrix jacchus* during necropsy. **A**: Extensive lesions with a necrotic appearance on the left thoracic limb; **B**: Necrotic lesions on the left pelvic limb; **C**: Infestation by ticks of the species *Rhipicephalus sanguineus* sensu lato in the ear; **D**: Intense parasitism by adult nematodes of *Primasubulura jacchi* in the small intestine

Externally, the animal presented necrotic skin lesions on the left thoracic and pelvic limbs, as well as a heavy tick infestation identified as *Rh. sanguineus* s.l., predominantly attached to the auricular region, axillary region, and dorsal cervical region. Internally, intense parasitism by *P. jacchi* was observed in the small intestine (Fig. 1).

**Fig. 2:**
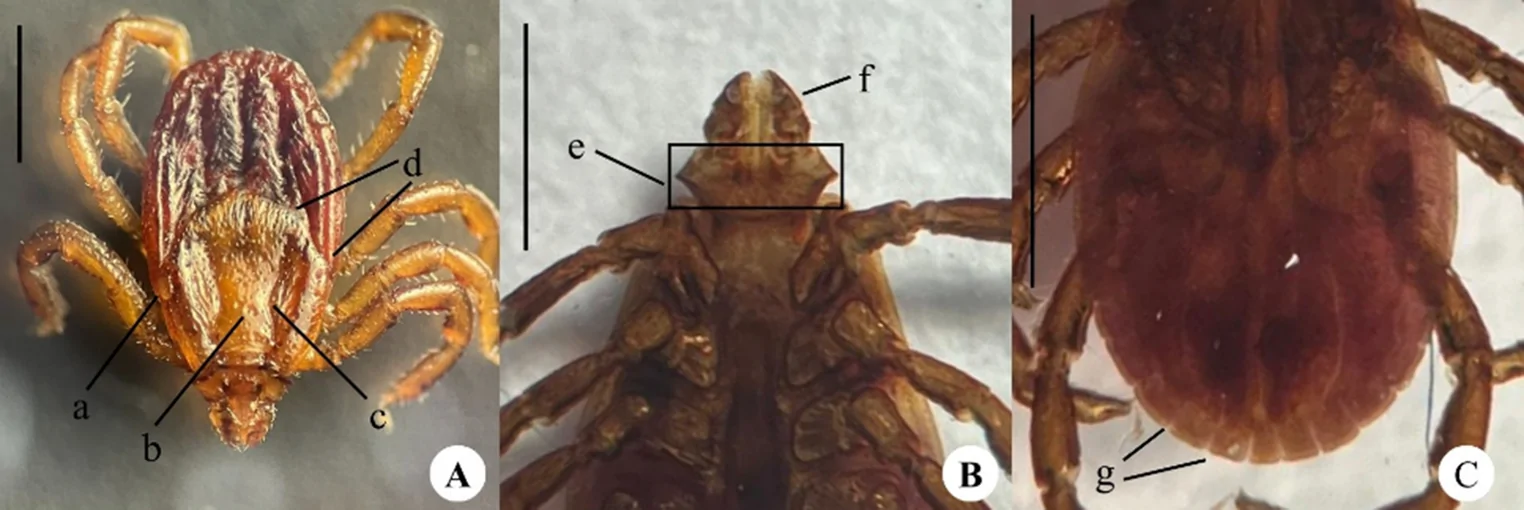
Specimens of *Rhipicephalus sanguineus* sensu lato found in *Callithrix jacchus* in a peri-urban area of Garanhuns, Pernambuco, Brazil. **A**: Dorsal view of a female *Rh. sanguineus*. **a**: Large and slightly convex eyes; **b**: Scutum with evident punctations; **c**: Cervical grooves on the scutum deep and distinct; **d**: Double concavity on both posterior margins of the scutum; **B**: Male *Rh. sanguineus* in cranioventral view; **e**: Basis capituli hexagonal; **f**: Palps short, generally similar in length to the basis capituli; **C**: Female *Rh. sanguineus* in caudoventral view; **g**: Festoons present. Scale bar = 1.0 mm

The ticks collected from the *C. jacchus* specimen (11 nymphs, 16 females, and 7 males) exhibited morphological characteristics consistent with *Rh. sanguineus* s.l. (Fig. 2) (Walker et al. 2000; Dantas-Torres et al. 2013). Morphologically, they present a reddish-brown coloration; large, slightly convex eyes positioned laterally at the level of the scutum; hexagonal basis capituli; short palps approximately the same length as the basis capituli; and well-defined festoons along the posterior margin of the idiosoma. Additionally, females showed a dorsal scutum with evident punctations, deep and well-defined cervical grooves, and a double concavity along the posterior margin of the scutum.

**Fig. 3:**
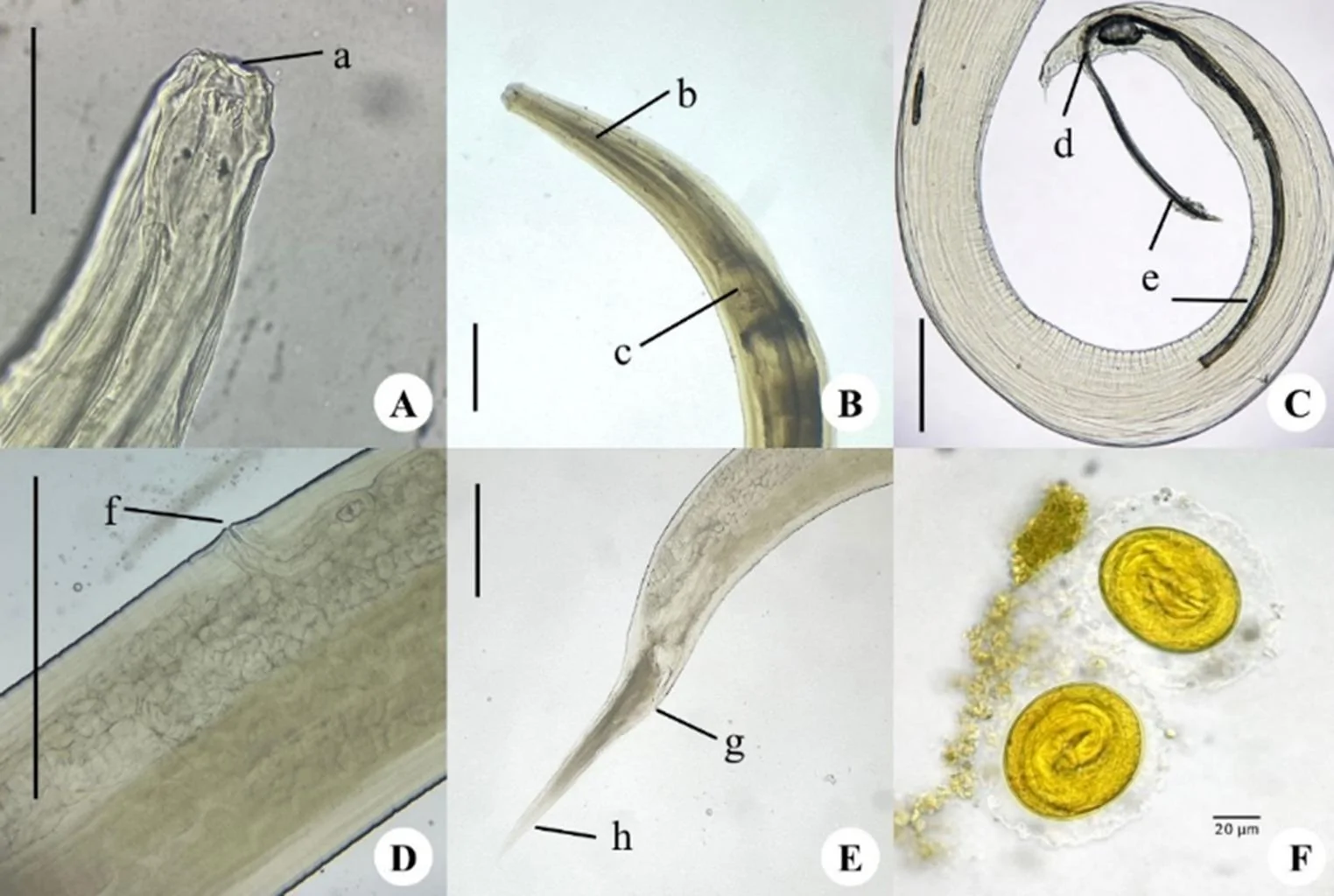
Specimens of *Primasubulura jacchi* collected from the small intestine of *Callithrix* *jacchus* in a peri-urban area of Garanhuns, Pernambuco, Brazil. **A**: Anterior extremity of female *P. jacchi*; **a**: Buccal capsule; Magnification 10×; **B**: Anterior extremity of female *P.jacchi*; **b**: Esophagus; c: Terminal esophageal bulb; Magnification 4×; **C**: Posterior extremity of male *P*. *jacchi*; **d**: Gubernaculum; **e**: Spicules; Magnification 10×; **D**: Median portion of female *P. jacchi*; **f**: Vulvar opening; Magnification 10×; **E**: Posterior extremity of female *P.jacchi*; **g**: Anus; h: Tail with tapered extremity; Magnification 10X; **F**: Eggs of *P. jacchi*; Magnification 40×. Scale bar = 0.5 mm

Eighty-six adult helminths were recovered, 67 females (77.9%; 95% CI = 68.0–85.3%) and 19 males (22.1%; 95% CI = 14.6–31.9%) recovered from the small intestine of *C. jacchus* showed morphometric features compatible with *P. jacchi*. Females (*n* = 10) measured 1.51–2.36 cm in length, with a mean of 1.88 cm (± 0.22), whereas males (*n* = 10) ranged from 0.85 to 1.20 cm, with a mean of 1.02 cm (± 0.14). Morphological characteristics (Fig. 3) included an anterior region with a cylindrical buccal capsule, no evident division between the buccal capsule and the esophagus, and the presence of a terminal esophageal bulb. In females, the vulva was located in the median portion of the body, and the posterior end had a tapered extremity. In males, the posterior end was curved, with an elongated gubernaculum and two spicules of similar size (Soares et al. 2024).

Coprological analysis revealed the presence of eggs measuring 66–75 μm in length (mean 70.1 μm ± 2.73) and 55–65 μm in width (mean 59.55 μm ± 3.36), with a thick, smooth shell (Fig. 3). Morphologically they were compatible with *P. jacchi*.

## Discussion and conclusion

Data presented herein indicated a high parasitism by *P. jacchi*, associated with the first record of *Rh. sanguineus* s.l. in *C. jacchus* in Northeastern Brazil, which expands knowledge of the parasitic fauna associated with marmosets in this region. These findings reinforce the role of urban and peri-urban environments in shaping host-parasite interactions, especially in species with high ecological plasticity, such as *C. jacchus*, which frequently occupies anthropized areas and maintains close contact with humans and domestic animals (Pinheiro and Pontes 2015; Andrade 2022).

The extensive necrotic skin lesions observed in the animal were of unknown origin, although trauma due to aggression cannot be excluded. In the present study, severe intestinal parasitism by *P. jacchi* and intense infestation by *Rh. sanguineus* s.l. were observed. Previous studies have suggested that heavy parasitic burdens and ectoparasite infestations may negatively affect body condition and contribute to inflammatory processes (Bernstein and Didier 2009).

*Primasubulura jacchi* is considered a common helminth in *Callithrix* spp. and other neotropical primates, with extensive reports across Brazilian regions covered by Atlantic Forest remanescents (Sales et al. 2010; Tavela et al. 2013; Dias et al. 2024). Transmission occurs when the primate ingests infected insects (e.g., orthopterans such as grasshoppers and crickets, or coleopterans such as beetles) containing infective larval stages (Melo and Pereira 1986; Sales et al. 2010). In free-ranging marmoset populations whose insects are part of the diet, the prevalence of *P. jacchi* is high.

Despite its high prevalence, it is not considered highly pathogenic, but rather an ecological indicator of the adaptation and invasive success of marmosets in new areas, since the host must be well adapted and actively exploiting food resources and microhabitats for the helminth’s life cycle to be maintained (Sales et al. 2010). Moreover, even in urbanized environments, the frequency of infected marmosets remains similarly high compared to wild populations, demonstrating that this nematode can efficiently maintain its life cycle in anthropized settings (Tavela et al. 2013).

*Rhipicephalus sanguineus* s.l. is associated with both intradomiciliary and peridomiciliary environments, where domestic dogs represent its main hosts, although this tick has also been reported in humans, other domestic and wild animals, including nonhuman primates (Lorusso et al. 2010; Martins et al. 2021). In addition, evidence regarding the maintenance of *Rh. sanguineus* developmental stages in natural environments remains limited. However, human activities are believed to influence the distribution of this tick in tropical regions such as Brazil (Lorusso et al. 2010; Nava et al. 2012), which makes the occurrence of *Rh. sanguineus* s.l. in *C. jacchus* in this region, particularly relevant.

Considering that the animal in the present study was rescued from a periurban area, this finding may suggest close contact between primate and human modified environments, such as buildings and areas with frequent tick infested dogs. Furthermore, in Northeastern Brazil, *Rh. sanguineus* s.l. has recognized medical and veterinary importance as a vector of pathogens such as *Ehrlichia canis*, *Anaplasma platys*, *Babesia vogeli* and *Hepatozoon canis* (Nogueira et al. 2024), which reinforces the need for a better understanding of interactions among wildlife, domestic animals, and anthropized environments under a One Health perspective.

The presence of *Rh. sanguineus* s.l. in nonhuman primates has previously been described in Central-Western Brazil in *Mico melanurus* (black-tailed marmoset), which was characterized as accidental parasitism without evidence of a stable natural ecological association between ectoparasite and host (Witter et al. 2016). However, this tick has also been reported in Northeastern Brazil parasitizing *Alouatta caraya* (black howler monkey) and *Ateles paniscus* (black spider monkey), as well as *Alouatta guariba clamitans* (brown howler monkey) in Southeastern region (Martins et al. 2021). Even if unusual, the occurrence of *Rh. sanguineus* s.l. in primates is not an isolated event and is not restricted to a single region or primate species.

Regarding marmosets of the family Callitrichidae, ectoparasitism by *Amblyomma aureolatum* and *Amblyomma sculptum* have been reported in the states of São Paulo and Minas Gerais, respectively. Additionally, *Haemaphysalis juxtakochi* and *Rh. sanguineus* s.l., have been reported parasitizing *Callithrix* spp. in the same region (Martins et al. 2021).

Overall, the results herein presented contribute to the understanding of the parasitic fauna associated with *C. jacchus* in urban and peri-urban environments of Northeastern Brazil. The identification of *P. jacchi*, a helminth frequently reported in marmosets, and the record of *Rh. sanguineus* s.l., an ectoparasite typically associated with canids and urban environments, expand current knowledge about the parasites that may occur in this species under conditions of intense environmental modification (Tavela et al. 2013; Dias et al. 2022). These findings reinforce the importance of regional parasitological surveys, especially in historically under-sampled areas, for characterizing primate parasitic diversity.

The findings of the present study should be analyzed from a One Health perspective, considering the increasingly close interface among wildlife, domestic animals, and humans in peri-urban environments. The identification of *Rh. sanguineus* s.l., a tick commonly associated with domestic dogs, in *C. jacchus* indicates the proximity between these hosts and reflects a scenario of habitat anthropization. Such interaction favors the circulation of parasitic agents among species and represents a risk not only to primates but also to domestic animals and humans sharing the same space. The possibility of pathogen spillover and amplification of certain zoonotic agents in a shared environment must also be considered (Narat et al. 2017; Gamble et al. 2023). The first record of *Rh. sanguineus* s.l. parasitizing *C. jacchus* in Northeastern Brazil significantly contributes to filling knowledge gaps regarding the parasitic fauna associated with neotropical primates in this region. These results highlight the importance of health monitoring of marmoset populations in urban environments, not only from a wildlife conservation perspective but also considering potential implications for animal and environmental health.

The present study reports the occurrence of *P. jacchi* and documents, for the first time, the presence of *Rh. sanguineus* s.l. parasitizing *C. jacchus* in Northeastern Brazil. These findings expand current knowledge of the parasitic fauna associated with marmosets in the region and highlight the influence of urbanized environments on the exposure of nonhuman primates to parasites typically associated with anthropized settings. The results reinforce the need for regional studies and health surveillance focused on *C. jacchus* populations in urban areas, particularly within a One Health framework.

## Supplementary information

Below is the link to the electronic supplementary material.

(PNG 1.25 MB)

## Data Availability

All data supporting the findings of this study are available within the paper.
