# Supplementary figures and images for: High parasitism by Primasubulura jacchi (Ascaridida: Subuluridae) and infestation of Rhipicephalus sanguineus sensu lato (Acari: Ixodidae) in Callithrix jacchus (Primates: Callitrichidae) in Northeastern Brazil
Source: Vet Res Commun. 2026 Jun 11;50(5):386. doi: 10.1007/s11259-026-11316-y (PMC13260178; doi:10.1007/s11259-026-11316-y)

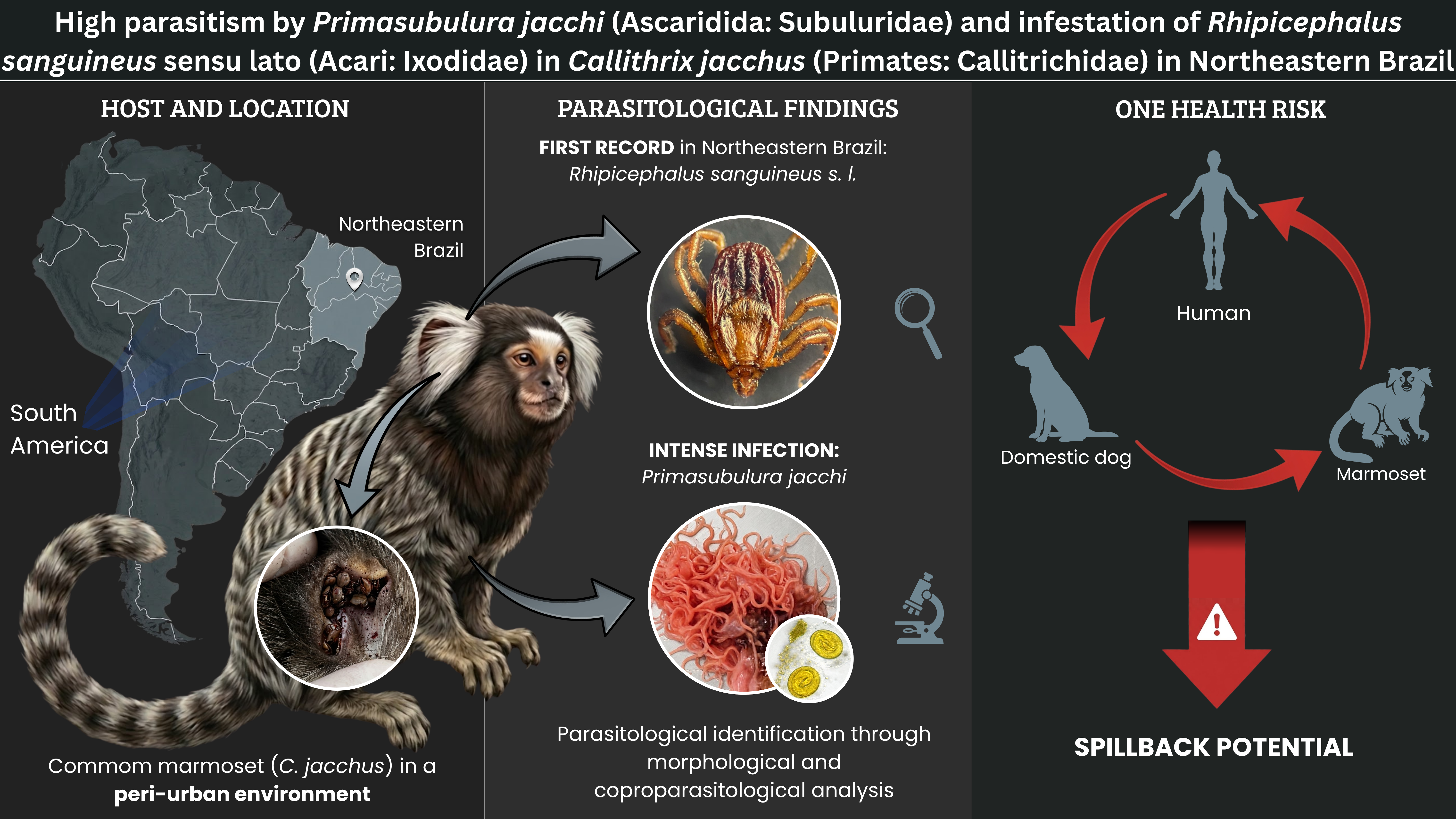

Supplement: Supplementary file 1 — (PNG 1.25 MB) [file 11259_2026_11316_Fig4_ESM.png]
